# Metagenome-assembled genome of a novel *Pseudoalteromonas* species from South Mid-Atlantic Ridge deep-sea water suggests potential for chitin degradation

**DOI:** 10.1128/mra.00189-25

**Published:** 2025-04-17

**Authors:** Zhiyi Wang, Yan Sun, Hongliang Wang, Juanli Yun, Wenbin Du

**Affiliations:** 1 State Key Laboratory of Microbial Diversity and Innovative Utilization, Institute of Microbiology, Chinese Academy of Sciences 85387, Beijing, Beijing, China; 2 College of Life Sciences and Medical School, University of the Chinese Academy of Sciences 617066 , Beijing, Beijing, China; 3 Microbial Resource and Big Data Center, Institute of Microbiology, Chinese Academy of Sciences 85387, Beijing, Beijing, China; 4 Chinese National Microbiology Data Center (NMDC), Beijing, China; 5 National Deep Sea Center, Ministry of Natural Resources, Qingdao, China; 6 School of Environmental Science and Engineering, Shaanxi University of Science and Technology 74618, Xi'an, Shaanxi, China; Montana State University, Bozeman, Montana, USA

**Keywords:** metagenome-assembled genome, *Pseudoalteromonas*, chitin degradation, deep-sea microbiology, South Mid-Atlantic Ridge

## Abstract

We report a high-quality metagenome-assembled genome (MAG) of a novel *Pseudoalteromonas* species recovered from deep-sea water of the South Mid-Atlantic Ridge. This MAG encodes key chitinase-related genes, suggesting potential involvement in chitin degradation and organic matter remineralization in the deep sea.

## ANNOUNCEMENT

Chitin is one of the most abundant biopolymers in marine ecosystems (1). Chitin-degrading bacteria play a crucial role in marine carbon and nitrogen cycling by breaking down chitin into bioavailable oligosaccharides and monomers, contributing to deep-sea biogeochemical cycles (2). Here, we report the draft MAG of a novel *Pseudoalteromonas* species from deep-sea water encoding chitinase genes, suggesting its potential role in chitin degradation and nutrient cycling in deep-sea ecosystems.

Deep-sea water was collected in February 2024 at 5,500 m depth from the South Mid-Atlantic Ridge (SMAR, 13°28′ W, 27°10′ S) using a CTD Rosette Sampler aboard the research vessel *Deep Sea No. 1*. Twenty liters of seawater was concentrated to 10 mL using a Large Volume Concentration Kit (Cat. No. CC01116, InnovaPrep, USA). The concentrated sample was diluted to approximately 1 × 10⁷ cells/mL in 2216E medium using a cell counting chamber (Cat. No. 177–112C, Watson, Japan) and then encapsulated as single cells into 30-pL droplets using a custom-built microfluidic device following laboratory-established protocols (3). Droplets were pooled and incubated at 25°C for 7 days and then demulsified with perfluorooctanol to collect enriched microbial cells.

Genomic DNA was extracted from 3 mL of demulsified droplet solution using the Bacterial DNA Extraction Kit (Cat. No. DZ314-03, FINDROP, China) following the manufacturer’s protocol. Sequencing libraries were prepared using the ALFA-SEQ DNA Library Prep Kit (Cat. No. NDI001E, FINDROP, China), and paired-end 150 bp reads were generated on the Illumina NovaSeq X Plus platform, yielding 37.59 Gbp of raw metagenomic data. Sequencing quality was assessed using Fastp (v0.23.2, default parameters) (4), and clean reads were assembled using SPAdes (v3.15.5) with the “--meta” option (5). MAGs were recovered using MetaWRAP (v1.3.2, default parameters) (6), and CheckM (v1.1.2) (7) identified eight high-quality MAGs (completeness >90%, contamination <5%). Taxonomic classification was performed via GTDB-Tk (v2.4.0, r220, default parameters) (8), and protein-coding sequences were predicted by PROKKA (v1.14.6, default parameters) (9). Predicted proteins were annotated against the KEGG database using DIAMOND BLASTp (v2.1.8, coverage >80%, identity >80%, e-value <1e-5) (10). Average nucleotide identity (ANI) was calculated using fastANI (v1.34) (11).

**Fig 1:**
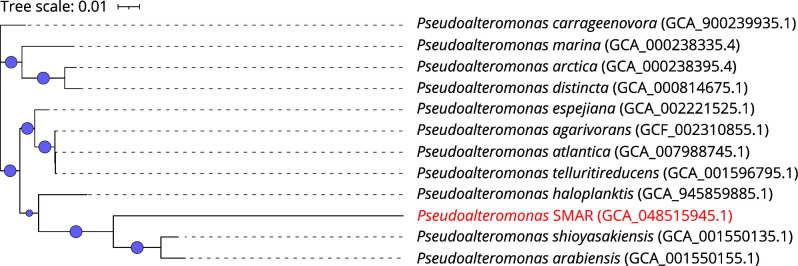
Maximum likelihood phylogenetic tree showing the relationship between *Pseudoalteromonas* SMAR (GCA_048515945.1) and closely related *Pseudoalteromonas* genomes. The tree was constructed using FastTree (v2.1.11) with the GTR model and 1,000 bootstrap replicates, based on the alignment of 120 conserved bacterial marker genes via GTDB-Tk. Node sizes correspond to bootstrap support values, with larger nodes indicating >75% support. *Pseudoalteromonas* SMAR is highlighted in red.

A MAG, designated *Pseudoalteromonas* SMAR (GCA_048515945.1), was identified, exhibiting high genome novelty and encoding genes associated with chitin degradation. GTDB-Tk classified it as an uncharacterized species within the genus *Pseudoalteromonas*, and its genomic features are summarized in Table 1. ANI analysis confirmed its novelty, with the highest similarity (78.37%) to *Pseudoalteromonas shioyasakiensis*. A phylogenetic tree illustrating its relationship to closely related species is presented in Fig. 1.

**TABLE 1:** Metagenome-assembled genome statistics for *Pseudoalteromonas* SMAR^*a*^

Parameter	Data
Sequence read archive accession number	SRR32475513 (GenBank) NMDC10019562 (NMDC)
BioSample accession number	SAMN46958406 (GenBank) NMDC20366030 (NMDC)
MAG accession number	JBLWMW000000000 (GenBank) NMDC60209776 (NMDC)
No. of reads for assembly	228553006
Completion (%)	98.13
Contamination (%)	0.168
G + C content (%)	45.54
N_50_ (bp)	88369
No. of contigs	60
Genome size (bp)	3947538
No. of CDSs	3467
No. of tRNA	37
No. of tmRNA	1
Encoded rRNA	Not detected

^*a*^ Note: GenBank and NMDC are public repositories for nucleotide sequence data maintained by NCBI and the National Microbiology Data Center of China, respectively.

Functional annotation of the *Pseudoalteromonas* SMAR suggests a putative role in chitin degradation. The genome encodes key enzymes, including chitinase (*chiA*) and N-acetylglucosaminidase (*nagZ*), which may facilitate the conversion of chitin into oligomers and subsequently into monomeric N-acetylglucosamine (GlcNAc) (2), a bioavailable source of carbon and nitrogen. A nearly complete chitin degradation pathway suggests potential involvement in organic matter remineralization within deep-sea environments.

## Data Availability

The MAG is available in GenBank under genome accession number JBLWMW000000000, associated with BioSample number SAMN46958406. The corresponding raw sequencing data are available under accession number SRR32475513.
